# Radiogenomics in brain, breast, and lung cancer: opportunities and challenges

**DOI:** 10.1117/1.JMI.8.3.031907

**Published:** 2021-06-18

**Authors:** Apurva Singh, Rhea Chitalia, Despina Kontos

**Affiliations:** University of Pennsylvania, Department of Radiology, Philadelphia, Pennsylvania, United States

**Keywords:** radiogenomics, prognostic signatures, precision medicine, cancer research

## Abstract

The field of radiogenomics largely focuses on developing imaging surrogates for genomic signatures and integrating imaging, genomic, and molecular data to develop combined personalized biomarkers for characterizing various diseases. Our study aims to highlight the current state-of-the-art and the role of radiogenomics in cancer research, focusing mainly on solid tumors, and is broadly divided into four sections. The first section reviews representative studies that establish the biologic basis of radiomic signatures using gene expression and molecular profiling information. The second section includes studies that aim to non-invasively predict molecular subtypes of tumors using radiomic signatures. The third section reviews studies that evaluate the potential to augment the performance of established prognostic signatures by combining complementary information encoded by radiomic and genomic signatures derived from cancer tumors. The fourth section includes studies that focus on ascertaining the biological significance of radiomic phenotypes. We conclude by discussing current challenges and opportunities in the field, such as the importance of coordination between imaging device manufacturers, regulatory organizations, health care providers, pharmaceutical companies, academic institutions, and physicians for the effective standardization of the results from radiogenomic signatures and for the potential use of these findings to improve precision care for cancer patients.

## 1 Introduction

A primary goal toward precision cancer care is the molecular characterization of disease using genomic and proteomic technologies.^1^^,^^2^ Although progress is being made, large-scale genome-based cancer characterization is not yet routinely performed for all cancers due to cost, turnaround time, and technical complexity.^3^^–^^5^ Additionally, molecular profiling is often limited in accuracy due to the heterogeneous nature of cancer. For example, in solid tumors, a histopathologic sample from a biopsied tumor may not fully reflect the anatomic, functional, and physiologic properties of the entire tumor.^6^^,^^7^ Moreover, the acquisition of tissue samples requires invasive procedures and repeated tissue sampling may not be feasible during treatment to monitor patient response.^8^

Medical imaging enables a non-invasive analysis of the functional and physiological properties of tumors, and the different available modalities are increasingly recognized for containing high-dimensional mineable data, which in turn can be used to improve medical decision making.^9^^,^^10^ Imaging can also help in characterizing peritumoral regions, which are not always surgically removed for molecular characterization^11^^,^^12^ and may convey information related to the tumor microenvironment.^13^^,^^14^ For example, imaging characteristics of tumors are increasingly being used to predict gene expression.^15^ Additionally, recent studies show that the molecular mechanisms of cancer are associated with specific imaging phenotypes.^16^ Thus medical imaging, earlier used primarily as a diagnostic tool, is now emerging as a key player in the field of personalized medicine for cancer by also providing prognostic and predictive information.^17^

Radiomics is an emerging field aiming to extract high-throughput quantitative data from routinely collected medical images.^10^^,^^18^^,^^19^ Typical first steps in a radiomic study involve the identification and segmentation of a region of interest (ROI). The segmentation may be carried out manually by human experts, or via semi- or fullyautomated segmentation algorithms.^20^^,^^21^ High-dimensional features subsequently extracted from these tumor regions are generally of two kinds: semantic and agnostic. Semantic features are mainly morphological features to describe the size, location, vascularity, spiculation, and necrosis of the lesion. Agnostic features are mathematically derived quantitative features, which can be further divided into three types: first-order statistical outputs (describing values within a single voxel), second-order statistical outputs (describing relationships between voxels), and higher-order statistical outputs (extracting patterns within an image through filter grids).^22^ Additionally, deep learning techniques can also be used to automatically extract high-level descriptive features from the tumor regions.^23^^,^^24^ These extracted radiomic signatures can then be used for a variety of purposes such as tumor classification and prediction of survival and response to therapy. Studies that find associations between imaging, genomic, and molecular data fall under the emerging field of radiogenomics.^25^ These studies aim to discover imaging surrogates for genomic signatures and to develop biomarkers leveraging the various data types used to characterize disease.^26^ These multidimensional biomarkers can then be used to predict survival and response to therapy and can play a crucial role in therapy personalization.

**Fig. 1:**
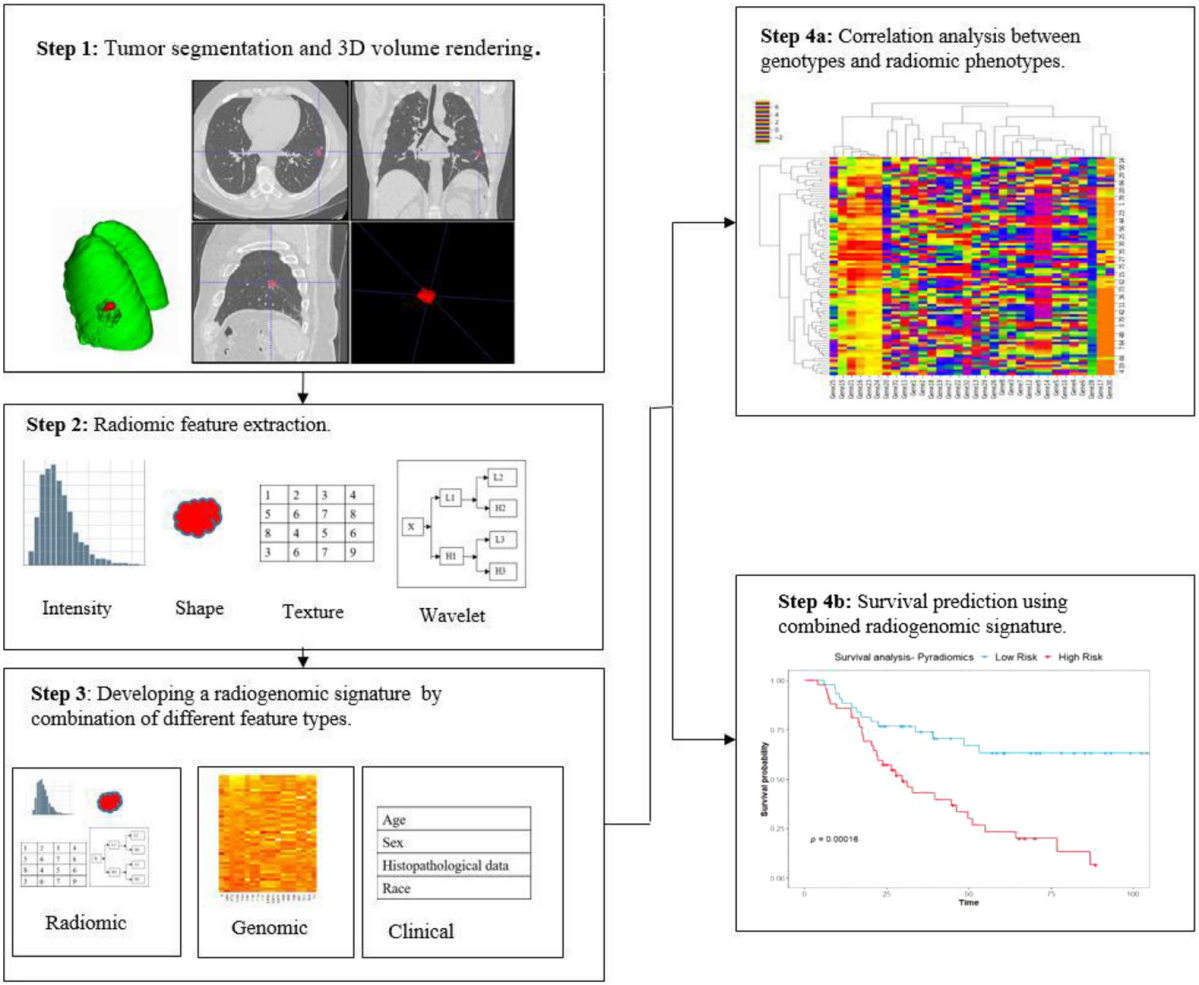
The basic steps in a radiogenomic study. Step 1: the tumor region is segmented and rendered as a 3D volume. Step 2: high-throughput radiomic features are extracted from the segmented tumor volume. Step 3: various feature types (clinical, radiomic, and genomic) are combined to develop a radiogenomic signature. Step 4(a): analysis of the correlations between radiomic phenotypes and genotypes to discover biologically significant radiomic signatures. Step 4(b): use of radiogenomic model to predict survival.

In this review paper, we aim to provide an overview of the radiogenomics studies conducted in cohorts of patients with cancer, focusing primarily on brain, breast, and lung carcinomas, to present a comprehensive perspective of progress within the field. An overview of the techniques used for the analysis of cancer sites where the radiogenomics field has largely been developed may assist in the development of related techniques in cancers where research is still in its nascent phases (Fig. 1).

The studies in the field of radiogenomics can be broadly divided into four overarching themes: identifying correlations between radiomic signatures and gene expression patterns, leveraging radiomic signatures to predict molecular subtypes of disease, combining radiogenomic models for patient outcome prediction, and identifying correlations between radiomic signatures and biological pathways. In the following sections, we review studies representing the state-of-the-art from these broad themes and conclude with what we believe are the current challenges as well as the opportunities for cancer radiogenomics research.

## 2 Correlations between Radiomic Signatures and Gene Expression Status

Studies exploring the correlation between radiomic signatures and gene expression status largely aim to understand the biologic basis of radiomic signatures using gene expression and molecular profile information. These studies may also aim to identify non-invasive radiomic biomarkers as surrogates for established genomic, prognostic, and predictive biomarkers. Significant correlations between radiomic features and expression patterns of target genes are established in the studies included below.

Beyond conventional radiomic signatures, relationships between deep learning techniques and genomic and molecular profile information have also been explored in studies of brain cancers. Deep learning techniques were used to extract 20 morphological features from contrast-enhanced and peritumoral edema regions and to establish a relationship between gene expression and imaging features. A neural network pretrained with an autoencoder and dropout had lower errors than linear regression in predicting tumor morphology features by an average of 16.98% mean absolute percent error and 0.0114 mean absolute error.^27^ Over the past few years, periostin (POSTN), a gene involved in cell survival and angiogenesis, has emerged as a marker for tumor progression and as a novel therapeutic agent in various types of human cancers. Causality between POSTN expression levels and radiomic signatures derived from magnetic resonance (MR) images and orthotopic xenografts (OX) was determined using a unique combination of skull stripping, brain-tissue focused normalization, and patient-specific normalization. Radiomic GLCM-based features predicted POSTN expression status in patients with an area under the curve (AUC) of 0.77 and in OX with an AUC of 0.92.^28^ In another study, 29 GBM patients were identified in the TCGA database that had corresponding MR imaging available through the TCIA and had overlapping mutations in either TP53, PTEN, or EGFR. Significant radiomic features for the three genotypes (TP53, PTEN, and EGFR mutated tumors) were identified. Consensus cluster analysis demonstrated similar correlation matrices for TP53 mutant versus wildtype radiomic texture features as for the corresponding gene expression results.^29^ Zinn et al. developed a clinically applicable analytical imaging method termed as “radiome sequencing” using patient data from TCGA/MD Anderson datasets. They derived 4800 MRI-derived texture features per tumor. A patient-specific genomic probability map was derived. Correlation between the imaging signature and EGFR amplification (AUC=0.86, p<0.0001), O6-methylguanine-DNA-methyltransferase methylation/expression (AUC=0.92) and glioblastoma molecular subgroups (AUC=0.88) was derived.^30^ In a study involving 22 GMB patients, semiautomatic tumor segmentation and feature extraction methods from MR images were used. Feature vectors were used to predict GBM phenotypes based on the nearest neighbor (NN) classifier (AUC=0.76).^31^ An MR imaging, messenger RNA (mRNA), and copy number variation (CNV) radiogenomic association map has led to the identification of MR traits associated with high-grade glioma biomarkers. Integration of MR imaging, mRNA, and CNV data resulted in the identification of individual genes and loci with correlated mRNA and CNV changes that are significantly associated with imaging features. Thirty-four unique genes were identified as being significantly correlated with at least one of the six imaging features.^32^

EGFR mutation status is becoming widely recognized as a useful biomarker for planning targeted therapy regimens in lung cancer patients. As such, many radiogenomic studies have focused on examining associations between radiogenomic signatures and EGFR mutation status. EGFR+, EGFR− and KRAS+ tumors were found to drive distinct radiographic phenotypes in a study with lung adenocarcinoma patients. The authors developed a radiogenomic signature consisting of features quantifying tumor intensity, texture, and shape features as well as wavelet and Laplacian of Gaussian features. This signature successfully discriminated between EGFR+ and EGFR− cases (AUC=0.69) and between KRAS+ and KRAS− cases (AUC=0.63).^15^ Texture features extracted from pretreatment CT and FDG PET-CT images were used to develop multivariate logistic regression models to predict EGFR mutations. Linear discrimination analysis was used to rank each texture feature individually in terms of its discriminatory importance. Logistic regression was used as a machine learning model to discriminate between EGFR mutant and wild type tumors with AUC, sensitivity, specificity, and accuracy of 0.87, 0.76, 0.66, and 0.71, respectively.^33^ Grossman et al. analyzed two independent cohorts of 262 North American and 89 European patients with lung cancer and found associations between radiomic imaging features, molecular pathways, and clinical factors. Intratumor heterogeneity features predicted activity of RNA polymerase transcription (AUC=0.62, p=0.03) and intensity dispersion was predictive of the autodegradation pathway of a ubiquitin ligase (AUC=0.69).^34^ In another study involving 404 NSCLC patients (243 training and 161 validation), radiomics features were extracted from preoperational non-contrast CT images of the tumor region. Correlations between EGFR mutation status and candidate predictors were assessed using the MWU test. The radiomic signature performed better at predicting EGFR mutation status (AUC=0.798) as compared to models built with clinical factors and conventional CT morphological features.^35^ Radiomic signatures were used to predict PD-L1 expression levels in a study performed with 399 NSCLC patients. Tumor regions were segmented from CT, PET, and PET/CT images and 24 radiomic features describing the tumor region were used to build the radiomic signature. For PD-L1 expression levels over 1%, the AUCs for the prediction accuracy were 0.86, 0.62, and 0.85 from the CT, PET, and PET/CT signatures, respectively.^36^ Gevaert et al. performed a radiogenomics analysis using 180 radiomic features derived from CT and PET/CT scans of 26 NSCLC patients. They found 243 statistically significant pairwise correlations between image features and metagenes. The prediction of metagenes in terms of the image features achieved an AUC between 0.59 and 0.84.^37^

The studies included in this section showed the correlations between radiomic signatures and gene expression status by demonstrating the use of radiomic signature in predicting tumor morphology, distinguishing GBM phenotypes, discriminating EGFR+, EGFR− and KRAS+ tumors, and differentiating specific PDL1 subtypes.

A summary of these studies can be found in Table 1.

**Table 1:** Studies analyzing correlations between radiomic signatures and gene expression status.

Disease	Image modality	Number of patients	Outcome	Results	Study
GBM	MRI	528	A supervised deep neural network pretrained with an autoencoder predicted tumor morphology features better than a linear regression model	Mean absolute error in prediction = 0.0114	Ref. 27
GBM	MRI	93 patients and 40 orthotopic xenografts (OX)	Assessment of causality between radiomic texture features from patients and xenografts and POSTN levels	AUC for causality: 0.77 in patients and 0.92 in OX	Ref. 28
GBM	MRI	29	Correlation cluster analysis demonstrated similar correlation matrices for TP53 mutant versus wildtype radiomic texture features as for the corresponding gene expression results	The gene expression profiles and heatmaps for mutational versus WT defining gene expression profiles (P<0.05) demonstrate a similar pattern as for genotype defining radiomic feature sets	Ref. 29
GBM	MRI	Automated pipeline with 4800 MRI features derived from tumor regions acquired from databases common to TCIA and TCGA	Correlation established between imaging signatures and the following: EGFR amplification, O6-methylguanine-DNA-methyltransferase methylation/expression, GBM molecular subgroups	AUC for correlation of imaging signature with:	Ref. 30
				1. EGFR amplification: 0.86	
				2. O6-methylguanine-DNA-methyltransferase-methlyation: 0.92	
				3. GBM molecular subgroups: 0.88	
GBM	MRI	22	GBM phenotypes distinguished based on the texture feature GLCM	AUC for phenotype discrimination = 0.76	Ref. 31
GBM	MRI	142	Construction of an EGFRvIII imaging signature characterizing tumor heterogeneity	Distinctive ability of imaging signature (AUC=0.88)	Ref. 32
Lung cancer	CT	763 (353 training and 352 validation)	Radiomic signature capturing tumor heterogeneity is successful in discriminating EGFR+, EGFR− tumors, and EGFR+, KRAS+ tumors	EGFR+, EGFR− (AUC=0.69); EGFR+, KRAS+ (AUC=0.63)	Ref. 15
Lung cancer	CT	149	Adenocarcinoma with wild-type EGFR was significantly associated with imaging signatures corresponding to larger and irregularly shaped tumors	Correlation between EGFR wild type gene expression and radiomic signature (p value=0.01)	Ref. 33
Lung cancer	CT	351	Radiomic signature of intratumor heterogeneity predicted the activity of RNA polymerase transcription and signature of intensity dispersion was predictive of the autodegradation pathway of a ubiquitin ligase	Prediction of:	Ref. 34
				1. Activity of RNA polymerase (AUC=0.62)	
				2. Autodegradation pathway of a ubiquitin ligase (AUC=0.69)	
Lung cancer	CT	404 (243 training and 161 validation cohorts)	Integrated model with radiomics signature and clinical features used to differentiate EGFR mutation status	AUC for validation cohort = 0.818	Ref. 35
Lung cancer	CT, PET, and PET/CT	399	Radiomic models built with features from CT, PET, and PET/CT images used to differentiate specific PD-L1 subtypes	For PD-L1 expression levels over 1%, AUCs for differentiating PDL1 subtypes using signatures from the following image types are:	Ref. 36
				1. CT: 0.86	
				2. PET: 0.62	
				3. PET/CT: 0.85	
Lung cancer	CT	26	Statistically significant pairwise correlations established between image features and metagenes	Correlation coefficient varies from 0.59 to 0.83	Ref. 37

## 3 Radiomic Signatures Used for Classification of Molecular Subtypes

Studies describing how radiomic signatures can be used to classify tumors based on their molecular subtypes are included below. These studies aim to non-invasively predict molecular subtypes to guide personalized decision making, especially for therapy selection and monitoring.

Lu et al. used MR phenotypes of patients diagnosed with glioblastoma and lower grade gliomas to classify five molecular subtypes based on isocitrate dehydrogenase (IDH) and 1p/19q genotypes with an AUC of 0.82.^38^ High-throughput features from T1-weighted, T2-weighted MR, and FLAIR images of 103 LrGG (lower grade glioma) patients (73: training and 30: validation) were extracted and SVM models were used to find optimal features for IDH and TP53 mutation detection. ANOVA and chi-square test were applied on clinical characteristics to confirm whether significant differences exist between three molecular subtypes. The highest AUC for detection of IDH and TP53 mutation was 0.87 for the validation cohort. The stratified accuracies of the three subtypes were 0.73, 0.72, and 0.70, respectively.^39^ Rathore et al. applied a radiomics approach to multiparametric MRI of de novo glioblastoma patients (n=208 discovery and n=53 replication cohorts). They discovered three distinct and reproducible imaging subtypes of glioblastoma with differential clinical outcome and underlying molecular characteristics, including IDH1 and EGFRvIII.^40^

Leithner et al. analyzed CE-MR images of 143 (91 training and 52 validation) breast cancer patients (luminal A, luminal B, and triple-negative subtypes). Radiomic features were extracted from the manually segmented tumor region and linear discriminant analysis followed by k-NN classification was used for separation of receptor status and molecular subtypes. The performance on the validation set was luminal A versus luminal B (AUC=0.79) and luminal B versus triple negative (AUC=0.77).^41^ In a study of the preoperative MR images from 275 breast cancer patients, 56 radiomic features were extracted from tumor region. Surrogate markers (ER, PR, and HER2) were used to categorize tumors by molecular subtype: ER/PR+, HER2− (luminal A); ER/PR+, HER2+ (HER2); ER/PR/HER2− (basal). The imaging features were shown to be associated with luminal A (p=0.0007) and luminal B (p=0.0063) molecular subtypes.^42^ Saha et al. analyzed preoperative images of a set of 922 invasive breast cancer patients (461 each in the training and validation cohorts). Machine-learning models built using radiomic features were used to predict the following molecular subtypes: luminal A (AUC=0.697) and triple negative breast cancer (AUC=0.654).^43^

The studies included in this section demonstrated the use of radiomic signatures in the classification of molecular subtypes. Radiomic signatures were used to classify IDH and 1p/19q status of gliomas, detect TP53 mutation, distinguish between luminal A, luminal B, and triple negative molecular subtypes. These studies suggest the ability for non-invasive representations of molecular phenotypes using radiomic data.

A summary of these studies can be found in Table 2.

**Table 2:** Studies analyzing correlations between radiomic signatures and molecular subtypes.

Disease	Image modality	Number of patients	Outcome	Results	Study
Glioma	MRI	214	Three-level machine learning model based on multimodal MR radiomics used to classify IDH and 1p/19q status of gliomas	AUC for detection of:	Ref. 38
				IDH: 0.922	
				1p/19q: 0.975	
Glioma	MRI	103	Support vector machine-based recursive feature elimination (SVM-RFE) adopted to find optimal feature for IDH and TP53 mutation detection	AUC for detection of:	Ref. 39
				IDH: 0.792	
				TP53:0.869	
GBM	MRI	261	Discovered three distinct and reproducible imaging subtypes of GBM with differential clinical outcome, including IDH1, O6-methylguanine DNA methyltransferase, and EGFRvIII	Analysis found subtype-specific radiogenomic signatures of EGFRvIII-mutated tumors, provided an *in vivo* portrait of phenotypic heterogeneity in GBM and pointed to the need for precision diagnostics	Ref. 40
Breast cancer	CE-MRI	143	Radiomic signature used to distinguish between luminal A, luminal B and triple negative molecular subtypes	AUC for:	Ref. 41
				Luminal A versus B (0.794)	
				Luminal B versus triple negative (0.771)	
Breast cancer	MRI	275	Multivariate analysis was used to determine associations between radiomic signature and luminal A, luminal B molecular subtypes	Correlation between imaging and luminal A (p=0.0007), luminal B (p=0.0063)	Ref. 42
Breast cancer	MRI	922	ML-based models used to predict: tumor surrogate molecular subtype, oestrogen receptor, progesterone receptor, and human EGF status	AUC for prediction of:	Ref. 43
				Luminal A (0.697)	
				Triple-negative breast cancer (0.654)	
				ER status (0.649)	
				PR status (0.622)	

## 4 Combined Radiogenomic Models for Outcome Prediction

Studies describing how the combination of radiomic and genomic features can improve the performance of predictive models are included below. These studies aim to evaluate the potentially augmented prognostic performance of combining complementary information encoded in radiomic and genomic data. A novel set of image texture features were computed from the joint intensity matrices (JIMs) of GBM regions in CE T1-weighted images and FLAIR sequences. JIM features in necrotic 176 and edema subregions were shown to be associated with survival (AUC 0.68 to 0.70). Combining JIMs, GLCM, and gene expression features improved the AUC value (0.78).^44^

Ashraf et al. analyzed dynamic contrast enhanced (DCE) MR images of 56 women (mean age, 55.6 years and age range, 37 to 74 years) diagnosed with estrogen receptor-positive breast cancer. In this study, a multiparametric imaging phenotype vector was extracted for each tumor using quantitative morphologic, kinetic, and spatial heterogeneity features. Multivariate linear regression was performed to test associations between DCE MR imaging features and tumor recurrence likelihood. There was a moderate correlation (P<0.001) between DCE MR imaging features and the recurrence score. Four dominant imaging phenotypes were detected, with two including only low- and medium-risk tumors.^45^ A similar study was conducted in which tumor size and enhancement texture, identified as good representatives of tumor heterogeneity, were combined with other radiomic features including size, shape, margin morphology, enhancement texture, and kinetic assessment to create a radiomic signature. This signature was then used to distinguish between good and poor prognosis, yielding AUC values of 0.88, 0.76, 0.68, and 0.55 for MammaPrint, Oncotype DX, PAM50 risks of relapse based on subtype and PAM50 risk of relapse based on subtype and proliferation, respectively.^46^ Tamez-Pena et al. calculated radiomic features quantifying tumor shape and texture to build models for predicting recurrence scores estimated using OncotypeDX and PAM50 gene expression microarrays. The model achieved AUCs of 0.83 and 0.78 for OncotypeDX and PAM50, respectively. The study indicates that molecular-based recurrence risk and breast cancer subtypes have observable radiographic phenotypes.^47^ Metabolic radiomic patterns of locally advanced breast cancer have also been shown to be associated with Ki67 expression and achievement of pCR to NAC and risk of recurrence.^48^

Nishino et al. performed a study involving EGFR mutant pulmonary adenocarcinoma patients given first line of treatment of Erlotinib or Gefitinib. They found 8-week CT tumor volume decrease to be an important biomarker for predicting overall survival when fitted as a continuous variable in a Cox model (P=0.01).^49^ A screen of 24 CT image features was performed on 172 NSCLC patients, followed by random forest variable selection incorporating the CT features plus six clinical-pathologic covariates to identify a biomarker associated with shorter (progression-free survival) PFS after therapy with ALK inhibitor criaotinib. Tumors with a disorganized vessel pattern had a shorter PFS with crizotinib therapy than tumors without this trait (11.4 versus 20.2 months, p=0.041).^50^

The studies included in this section demonstrated the use of combined radiogenomic models for outcome prediction. Radiogenomic phenotypes were used to classify tumors at low versus high risk of recurrence, predict Oncotype DX and PAM50 recurrence scores, discriminate ALK+ tumors with shorter progression-free survival. These studies suggest the augmented prognostic and predictive performance of leveraging the complementary information provided by radiomic and genomic data.

A summary of these studies can be found in Table 3.

**Table 3:** Studies related to the survival prediction performance of radiogenomic models.

Disease	Image modality	Number of patients	Outcome	Results	Study
GBM	MRI	73	Texture features computed from the JIMs of GBM subregions are combined with GLCM and gene expression features are used to build a radiogenomics signature that classifies patients into short or long survival groups	Classification accuracy AUC=0.78	Ref. 44
Breast cancer	DCE-MRI	56	Multiparametric imaging phenotype vector extracted from tumor regions was used to classify tumors at low versus medium versus high risk of recurrence	Classification accuracy AUC=0.82	Ref. 45
Breast cancer	MRI	84	MR imaging phenotype used to evaluate risk of recurrence relative to multigene assay classifications	Prediction accuracy AUC: MammaPrint-0.88	Ref. 46
				Oncotype DX: 0.76	
				PAM50: 0.68	
Breast cancer	Digital mammograms	71	Radiogenomics signature used to predict Oncotype DX and PAM50 recurrence scores	Prediction accuracy AUC:	Ref. 47
				Oncotype DX: 0.83	
				PAM50: 0.78	
Breast cancer	FDG-PET/CT	73	Metabolic radiomic signature is associated with Ki67 expression achievement of pathologic complete response NAC and risk of recurrence	Metabolic radiomics patterns of LABC are associated with Ki67 expression (statistically significant p value<0.01)	Ref. 48
NSCLC	CT	44	Association between 8-week tumor volume decrease and survival	Association with overall survival (Cox model p value-0.01)	Ref. 49
NSCLC	CT	172	Radiogenomic biomarker used to discriminate ALK+ from non-ALK tumors and identify patients with a shorter PFS	Discriminatory power AUC=0.894	Ref. 50

## 5 Radiomic Signatures Correlated with Biological Pathways

Finding associations between radiomic signatures and biological pathways can help ascertain the biological significance of radiomic phenotypes and improve our understanding of what each radiomic phenotype represents. Studies describing how radiomic signatures can be correlated with biological pathways are included below. Yeh et al. established correlations between radiomic morphology features and various biologic pathways including replication, proliferation, immune signaling, extracellular signaling, metabolic, catabolic, JAK/STAT, and VEGF pathways.^51^ Certain clinical and imaging features derived from ALK/ROS1/RET fusion-positive lung adenocarcinoma patients were found to be good discriminators of fusion-positive and fusion- negative lung adenocarcinomas. A total of 539 pathologically confirmed lung adenocarcinomas were included in the study. The fusion-positive tumor prediction model was a combination of younger age, advanced tumor stage, solid tumor on CT, higher values for SUVmax and tumor mass, lower values for kurtosis, and inverse variance on 3-voxel distance than those of fusion-negative tumors (sensitivity and specificity, 0.73 and 0.70, respectively).^52^ Radiogenomic correlations were established between semantic image features and metagenes in NSCLC patients, which was also representative of canonical molecular pathways. A cohort of 113 patients with preoperative CT data and tumor tissue was used for the study. The authors recorded 87 semantic image features. Next, total RNA was extracted from the tissue and analyzed. RNA sequencing analysis resulted in 10 metagenes that capture a variety of molecular pathways, including the epidermal growth factor (EGF) pathway. A radiogenomic map was created with 32 statistically significant correlations between semantic image features and metagenes.^53^

The studies included in this section analyzed the correlations between radiomic features and biological pathways. Radiomic signatures were shown to be significantly correlated with breast cancer gene sets, used to discriminate fusion-positive tumors, predict autodegradation pathway of a ubiquitin ligase. These studies suggest that understanding the biologic and molecular underpinnings of radiomic features can allow for a non-invasive understanding of tumor behavior.

A summary of these studies can be found in Table 4.

**Table 4:** Studies analyzing correlations between radiomic features and biological pathways.

Disease	Image modality	Number of patients	Outcome	Results	Study
Breast cancer	DCE-MR	47	Automated, quantitative radiomics platform used on breast MR imaging for inferring underlying activity of clinically relevant gene pathways derived from RNA sequencing of invasive breast cancers	Tumors with higher expression levels of JAK/STAT and VEGF pathways had more intratumor heterogeneity. Metabolic and catabolic pathways also had associations with image-based features	Ref. 51
Lung cancer	CT and PET	539	Radiomic signature used to discriminate fusion-positive tumors	Discriminatory ability AUC=0.73	Ref. 52
NSCLC	CT	113	Radiogenomics map links semantic image features to metagenes	32 significant pairwise associations between quantitative image features and metagenes	Ref. 53

## 6 Opportunities and Challenges for Radiogenomics

Medical imaging can provide a non-invasive approach for tumor characterization and is routinely acquired throughout patient care. Additionally, it can capture characteristics of both the whole-tumor and the surrounding peritumoral area as opposed to analyses performed on biopsied tissue alone, which can often be limited by tumor sampling. Radiomic features derived from tumor images, when combined with cellular and molecular pathway information derived from gene expression assays, can offer a well-rounded characterization of the tumor region. Such combined analyses can advance personalized medicine across a variety of cancer types.

Imaging features extracted from tumor regions of interest are influenced by a variety of factors. The changes in scanner acquisition protocol parameters, such as resolution, slice thickness, reconstruction kernel, field-of-view, or other factors including patient movement during imaging, changes in treatment, and varying image acquisition protocols across institutions can all affect the resulting extracted features. This poses a challenge to feature robustness and reproducibility. Recent studies have explored feature reproducibility and robustness across heterogeneous data. A unique computed tomography data cohort of same-day repeated scans allowed for image reconstruction of each scan at six imaging settings, varying slice thicknesses (1.25, 2.5, and 5 mm) and reconstruction algorithms (sharp and smooth). Using this, two experiments were performed: using repeat scans reconstructed at identical imaging settings (six settings in total) and using repeat scans reconstructed at the same slice thickness with different algorithms (three settings in total). Interchanging smooth and sharp reconstruction algorithms were found to reduce feature reproducibility.^54^ In another reproducibility study, CT scans were obtained at different dose levels, section thicknesses, kernels, and reconstruction algorithm settings.^55^ Only intensity, shape, and texture radiomic features were found to be reproducible across the settings. A radiomics model for the prediction of EGFR mutation status was developed by selecting the optimal standard of care CT image from the following four combinations: two slice thicknesses (thin: 1 mm and thick: 5 mm) and two convolution kernels.^56^ Significant differences in the survival prediction model performance were observed in the features obtained from thick and thin CT slices. Hassan et al. investigated the impact of pitch, dose, and reconstruction kernel on CT radiomic features and introduced correction factors (NPS peak fraction and ROI maximum intensity) to reduce feature variability introduced by reconstruction kernels. Percentage improvements in robustness of 19 features were in the range of 30% to 78% after corrections.^57^ In another study performed to investigate the effect of variability in x-ray tube current on quantitative CT radiomic features, the credence cartridge radiomics phantom was scanned 12 times, varying the tube current while keeping the other parameters constant. The study concluded that variable x-ray tube current is unlikely to have a large effect on radiomic features extracted from CT images of texture objects such as tumors.^58^ Ger et al. combined PET scans of a Hoffman brain phantom acquired from GE Discovery 710, Siemens mCT, and Philips Vereos scanners in their study. A standard-protocol scan was acquired and then each parameter that could be changed was altered individually. To determine the impact of each parameter on the reliability of each radiomic feature, the ICC (intraclass correlation coefficient) was computed. When the pixel size was resampled prior to feature extraction, all features had good reliability (ICC>0.75) for the field of view and matrix size. They concluded that caution must be used when combining patients scanned on equipment from different vendors.^59^ Another study aimed to assess the agreement among radiomic features when computed by several groups using different software packages under very tightly controlled conditions.^60^ Nine common quantitative imaging features were selected for the comparison. The coefficient of variation (CV) was calculated across software packages for each feature on each object. Five of the nine features showed excellent agreement with CV <1%. The study highlights the value of feature definition standardization. Lo et al. investigated the effects of dose level and reconstruction method on density and texture-based features computed from lung CT nodules. A measure Q was introduced, to assess the stability of features across different conditions. Histogram mean was found to be the most robust feature in the study. The authors concluded that variation in density and texture features should be considered if a variety of dose and reconstruction conditions are being used.^61^

The studies mentioned above show the need to account for heterogeneity in image acquisition parameters to build robust radiogenomic signatures. Publicly available datasets, such as those common to TCIA and TCGA, can be used as validation sets in examining the effect of normalization of differences in radiomic features arising from variation in image acquisition parameters on the radiogenomic signatures built from them.

Coordination between imaging device manufacturers, regulatory organizations, health care providers, academic institutions, biopharmaceutical companies, and practicing physicians is important for effective validation and standardization of the results of radiogenomic studies.^62^ Following this vision, the National Cancer Institute initiated the quantitative imaging network (QIN) aimed to evaluate imaging methods measuring response to cancer therapy. QIN aims to create teams of oncologists, radiologists, medical physicists, and computer and informatics scientists partnered with industry representatives to develop retrospective and prospective databases with clinical outcome data. The intent is to evaluate and optimize current quantitative imaging methods and to develop newer methods to measure the response to drug and radiation therapy.^63^ The overall goal is to create an array of imaging platforms for different targeted organ systems and extend them to academic and industry-based researchers for evaluation of their techniques. Following this goal, quantitative imaging biomarker alliance was established at the annual Radiological Society of North America session in 2007.^64^ This initiative seeks to combine stakeholders such as national regulatory agencies to collectively determine validation methods for imaging biomarkers. A similar step was taken by The Imaging Biomarker Standardization Initiative, which validated consensus-based reference values for 169 radiomics features, thus enabling calibration and verification of various radiomics software.^65^ Once a quantitative imaging biomarker has been accepted by the community, it may then be utilized to generate more convincing study results. Although these initiatives have been developed for radiomics analysis, applying them to standardize imaging signatures employed in radiogenomics analyses can help determine their applicability to radiogenomics studies in the future.

In closing, we summarize the major takeaways from our review of the literature in the field of radiogenomics. The field of radiogenomics holds a lot of promise. Radiogenomic studies can help toward understanding the biologic basis of radiomic phenotypes by leveraging gene expression and molecular profile information. They may also show correlations between radiomic signatures, biological pathways, and gene expression status and help establish radiomic biomarkers as surrogates for genomic prognostic biomarkers. As shown in previous studies, radiomics and genomics features improve the performance of survival prediction models in combination with clinical information. The need to account for heterogeneity in image acquisition parameters to build robust radiomic signatures is increasingly being recognized. The development of a robust radiomic signature will further contribute to the robustness of the radiogenomic analysis performed using them. Coordination between clinicians, researchers, and manufacturers will play a major role in setting up a standard study pipeline. As such, consistent, meaningful, and accurate interpretations of patient data derived from genomic, proteomic, and radiomic analyses can improve patient care toward the goal of precision medicine.
